# Brain Imaging and Cognitive Deficits in Psychiatric Disorders

**DOI:** 10.3390/biomedicines12122888

**Published:** 2024-12-18

**Authors:** Yudan Ding, Wenbin Guo

**Affiliations:** Department of Psychiatry, National Clinical Research Center for Mental Disorders, and National Center for Mental Disorders, The Second Xiangya Hospital of Central South University, Changsha 410011, China; dandandding@gmail.com

Over the last three decades, various neuroimaging techniques, such as magnetic resonance imaging (MRI), functional MRI, and positron emission tomography (PET), have played an essential role in improving our understanding of the pathogenesis of neuropsychiatric disorders. A great number of structural and functional studies of the brain have identified regions and networks associated with various clinical symptoms and cognitive impairments, in disorders like schizophrenia, bipolar disorder, and major depressive disorder (MDD), and have shown strong potential as biomarkers to aid in diagnosis and treatment planning [1].

However, despite notable progress, significant gaps remain in the understanding of the clear mechanisms of these diseases, and the translation of key findings into clinical practice has been minimal. One of the barriers in the field is the inconsistency of findings across studies, which might be attributed to factors such as differences in patient populations, imaging techniques, and analytical methods. This variability makes it difficult to establish reliable neuroimaging biomarkers for clinical application. Moreover, most neuroimaging studies are cross-sectional, offering only a snapshot of brain activity or structure at a single point in time, which fails to clarify how abnormalities develop over time. In addition, the biological complexity of psychiatric disorders such as schizophrenia makes it unlikely that they can be adequately assessed with just one or two biomarkers. Instead, combining multiple imaging parameters with genetic and molecular data may provide a more comprehensive understanding of the biological processes underlying clinical symptoms and cognitive impairments. However, this kind of integration remains limited [2].

## 1. An Overview of Published Articles

This Special Issue focuses on the roles of brain activity and the neuropathological mechanisms underlying cognitive deficits as well as clinical symptoms in neuropsychiatric disorders, aiming to address important conceptual and methodological questions. In the following paragraphs, we briefly describe the five published articles and encourage readers to explore these interesting studies in detail to gain a deeper understanding of the advances made in this area.

Fan Zebin’s [3] article identified hypoactivity in the prefrontal cortex, visual cortex, and insula in patients with first-episode MDD during self-face recognition tasks compared to healthy individuals. This finding suggests the involvement of these brain regions in the pathophysiology of negative self-referential cognition, which plays a key role in the onset, maintenance, and recurrence of depressive episodes. This study may also inspire the development of new therapeutic targets for adults with first-episode MDD.

The article by Chen et al. [4] conducted a Mendelian randomization study, examining causal associations between five genetically predicted sleep traits (morningness, ease of getting up, insomnia, short sleep, and long sleep) and the cortex structure. This study provided new evidence for the effect of sleep habits on cortical changes, which in turn affect brain function. The integration of genetic and neuroimaging data in this study highlights the potential for multimodal approaches to understanding psychiatric disorders.

The study by Yan et al. [5] on panic disorder demonstrated that aberrant activity in the fear network, including the fronto-limbic-insula and temporal–occipital–parietal areas, partially normalized after pharmacotherapy. This study provided valuable longitudinal insights into how brain activity changes following treatment, contributing to a better understanding of panic disorder management.

The research by Huang et al. [6] focused on the prevalent sleep disorder obstructive sleep apnea (OSA), aiming to investigate the relationship between sleep characteristics, cognitive impairments, genetic factors, and brain structure and function in OSA. A key highlight of this study is that it combined MRI with transcriptomic analysis and sheds light on the intrinsic molecular genetic mechanisms underlying alterations in brain structure and function among individuals with OSA.

The focus of Marino’s work [7] is the cerebellum, a region traditionally associated with motor coordination but now recognized for its role in cognitive performance. They investigated brain functional interactions within the cerebellum and their relationship with cognitive deficits in schizophrenia. Interestingly, their findings suggested a gradient of language-related cerebellar connectivity alterations that corresponded with hallucination vulnerability in patients with schizophrenia.

## 2. Future Research Directions

As previously mentioned, although thousands of neuroimaging studies on psychiatric disorders are published each year, their clinical utility remains limited. A fundamental challenge is the heterogeneity of these disorders. The diagnostic criteria for psychiatric disorders still rely on clusters of symptoms and signs, which creates difficulties in differentiating between disorders in clinical practice. Symptom overlap is common, and many conditions share similar etiologies and structural or functional brain alterations [8]. To address this issue, future studies might benefit from increasing the variability of samples within study cohorts. Additionally, a multimodal framework could serve as a bridge to overcome current limitations. Collaborative neuroimaging studies with sufficiently powered genetic and epigenetic data are essential [1], and it would be advantageous to incorporate biosystems approaches such as proteomics, metabolomics, or blood transcriptomics [9,10]. Another challenge is that most studies rely on case–control designs, comparing patients with a specific psychiatric disorder to healthy participants. While large-scale imaging datasets like ENIGMA and REST-meta-MDD are invaluable for identifying robust group differences and uncovering novel brain–behavior relationships, this approach is less effective in developing clinically applicable biomarkers [1]. Abi-Dargham et al. proposed that studies should be structured from the beginning with the specific aim of developing biomarkers for a particular condition, focusing on the appropriate population. In so doing, psychiatry could shift away from broad, non-specific big data studies and move toward a more targeted approach, similar to the pipeline used in drug development [1].

## 3. Conclusions

This Special Issue highlights the ongoing advancements in neuroimaging and their potential to improve our understanding of the neural mechanisms underlying psychiatric disorders. While the five published articles offer significant contributions, especially in identifying brain regions and networks associated with cognitive impairments and clinical symptoms, many challenges remain. The future of neuroimaging lies in collaborative, cross-disciplinary efforts that will bridge these gaps and pave the way for more effective diagnostic and treatment strategies in psychiatric care.
